# Supplementary material for: Stimulation of Potent Humoral and Cellular Immunity via Synthetic Dual-Antigen MVA-Based COVID-19 Vaccine COH04S1 in Cancer Patients Post Hematopoietic Cell Transplantation and Cellular Therapy
Source: Vaccines (Basel). 2023 Sep 15;11(9):1492. doi: 10.3390/vaccines11091492 (PMC10538048; doi:10.3390/vaccines11091492)
Supplement: Supplementary file 1 [file vaccines-11-01492-s001.zip › vaccines-2573932-supplementary.pdf]

Supplementary Material

Supplementary Tables

Table S1. Patient characteristics and treatments

						COVID-19 SOC vaccine			SARS-CoV-2 infection		
**Patient (COH-)**	**Age[1]**	**Sex**	**Type of therapy**	**Diagnosis**	**Days from therapy to COH04S1**	**Donor**	**Pre-therapy**	**Post-COH04S1 (day of study)**	**Pre-therapy**	**Post-COH04S1 (day of study)**	**Therapy**
**201**	38	M	Allo-HCT	AML	118	2 doses	none	Pfizer (150)	none	none	
**202**	55	M	Allo-HCT	Myelofibrosis	171	N/A	2 doses, Pfizer	none	none	X (53)	R
**203**	67	F	Auto-HCT	MM	148	/	2 doses, Moderna	Moderna (69)	none	none	
**204**	52	F	Auto-HCT	MM	130	/	2 doses, Pfizer	none	none	X (94)	P
**205**	65	F	Allo-HCT	CMML	149	N/A	2 doses, Pfizer	none	none	none	
**206**	73	M	CAR-T cells	Lymphoma	247	/	2 doses, Moderna	Moderna (139)	none	none	
**207**	69	M	Auto-HCT	MM	103	/	2 doses, Pfizer	none	none	none	
**208**	61	F	Allo-HCT	AML	179	N/A	2 doses, Pfizer	none	none	none	
**209**	45	M	Allo-HCT	AML	235	none	none	none	none	X (145)	R
**210**	49	F	Allo-HCT	AML	198	N/A	2 doses, Moderna	none	none	none	
**211**	41	F	Auto-HCT	Hodgkin's lymphoma	106	/	2 doses, Pfizer	none	none	none	
**213**	47	F	Auto-HCT	Amyloidosis	238	/	2 doses, Pfizer	none	none	X (116)	none
**215**	47	F	Auto-HCT	MM	124	/	2 doses, Moderna	none	none	none	

[1]= years, SOC= standard of care, M= male, F= female, Allo= allogeneic, Auto= autologous, HCT= hematopoietic cell transplant, AML= Acute myeloid leukemia, MM= Multiple myeloma, CMML= Chronic myelomonocytic leukemia, N/A= not available, /= not applies, Pfizer= Comirnaty®, Moderna= Spikevax®, R= Remdesivir®, P= Paxlovid®.

Supplementary Table S1 (continued)

Patient (COH-)	IVIG	Evusheld (days before study)	GVHD (gr 3 to 4)	Steroids	Anti-neoplatic	mAb	TK/ Proteasome inhibitors	Immuno-modulatory drugs	Others
**201**			none						
**202**			none						
**203**			N/A						
**204**	X		N/A	Lo	Bortezomib	Daratumumab, Teclistamab			Radiation
**205**			none				Itacitinib	Tacrolimus, Sirolimus	
**206**	X		N/A						
**207**			N/A	Lo		Denosumab	Kyprolis	Revlimid	
**208**	X		cGVHD	Hi			Itacitinib, Ruxolitinib	Tacrolimus, Sirolimus	
**209**			None				Midostaurin		
**210**			cGVHD	Lo			Ruxolitinib	Tacrolimus, Sirolimus	
**211**		-108, -54	N/A						
**213**		-187	N/A						
**215**		-125	N/A					Revlimid	

IVIG= Intravenous immune globulin, GVHD= Graft versus host disease, gr= grade, c= chronic, N/A= not available, Hi= high dose (>0.5 mg/Kg/day), Lo= low dose (<0.5 mg/Kg/day), mAb= monoclonal antibody.

Table S2. Humoral response geometric mean and geometric mean fold increase by study day

			**Geometric Mean (GM)**				**Geometric Mean Fold Rise (GMFR)**			
				95% CI				95% CI		
**Parameter**	Day	N	Est GM	LL	UL	p-value	Est GMFR	LL	UL	p-values
**ELISA S**	0	13	90.2	17.7	458.8	0.0001	NA	NA	NA	NA
	28	13	1242.6	263.0	5870.9	<0.0001	13.8	3.6	52.9	0.0011
	56	12	3272.5	1114.1	9612.6	<0.0001	35.2	7.5	166.4	0.0004
	84	5	12046.6	4461.9	32524.4	<0.0001	39.0	1.9	778.8	0.0274
	120	5	851.1	38.3	18919.7	0.0038	21.3	0.6	727.4	0.0739
	140	4	3887.5	1649.4	9162.5	0.0001	7.6	0.4	145.3	0.1167
	180	6	11727.5	4520.2	30426.5	<0.0001	21.6	1.4	339.8	0.0353
**ELISA RBD**	0	13	45.8	9.9	212.1	0.0002	NA	NA	NA	NA
	28	13	516.4	96.8	2753.8	<0.0001	11.3	1.8	68.9	0.013
	56	12	1267.6	475.3	3380.7	<0.0001	26.5	4.9	142.1	0.0013
	84	5	4384.5	1038.4	18512.7	0.0001	53.4	0.4	6571.3	0.0834
	120	5	443.4	28.6	6862.6	0.0035	13.4	0.7	272.3	0.0753
	140	4	1639.3	349.8	7683.6	0.0006	13.1	0.1	3248.9	0.2341
	180	6	5510.9	1505.7	20169.2	<0.0001	38.4	0.6	2309.1	0.0707
**ELISA N**	0	13	2.3	1.2	4.3	0.0151	NA	NA	NA	NA
	28	13	5.7	1.7	19.1	0.0087	2.5	0.7	9.3	0.1586
	56	12	20.6	5.5	77.5	0.0004	8.8	1.6	47.1	0.0159
	84	5	211.2	37.9	1178.5	0.001	73.1	3.1	1704.3	0.0194
	120	5	7.3	0.6	96.0	0.0993	2.9	0.1	71.0	0.4159
	140	4	57.3	5.7	575.3	0.0113	23.8	0.6	1012.2	0.0744
	180	6	112.1	10.7	1168.8	0.0035	60.4	3.4	1080.7	0.0147
**NAb Wuhan**	0	13	94.2	20.5	434	<0.0001	NA	NA	NA	NA
	28	13	1119.9	236.9	5293.8	<0.0001	11.9	2.2	65	0.008
	56	12	2466.7	824.8	7377	<0.0001	26.1	4.2	161.9	0.0023
	84	5	3254.1	619.8	17083	0.0002	13.2	0.1	1411.3	0.1999
	120	5	605.0	20.5	17882	0.0063	8.9	0.1	635.7	0.229
	140	4	1977.5	451.6	8658.7	0.0005	4.9	0.1	286.3	0.3029
	180	6	3682.0	1807.9	7498.8	<0.001	11.5	0.3	437.0	0.1454
**NAb Beta**	0	13	69.3	17.9	268.3	<0.0001	NA	NA	NA	NA
	28	13	812.1	177.1	3723.1	<0.0001	11.7	2.9	47	0.0023
	56	12	1764.8	422.3	7376.2	<0.0001	21.7	4.5	105.2	0.0013
	84	5	4368.2	689.6	27669	0.0002	24.0	0.4	1435.4	0.0973
	120	5	280.1	8.6	9097.1	0.0109	5.1	0.2	132.3	0.2356
	140	4	3659.6	1048.5	12773.5	0.0002	14.2	0.3	756.8	0.1232
	180	6	3086.6	1816	5246	<0.0001	11.8	0.4	310.3	0.1097
**NAb Delta**	0	13	92.4	21.8	391	<0.0001	NA	NA	NA	NA
	28	13	928.8	179.7	4798.9	<0.0001	10.1	2.1	48.9	0.0079
	56	12	2249.4	634.9	7968.7	<0.0001	23.0	4.3	123.9	0.0018
	84	5	2409.8	387.1	15000.9	0.0003	11.6	0.2	703.1	0.1728
	120	5	271.8	11.0	6736.3	0.0084	3.2	0.1	135.4	0.4422
	140	4	1125.2	303.7	4168.5	0.0004	3.7	0.1	206.9	0.3826
	180	6	1953	1067.5	3573.3	<0.0001	6.2	0.2	157	0.2064
**NAb Omicron BA.1**	0	13	35.9	10.9	118.5	<0.0001	NA	NA	NA	NA
	28	13	331.2	76.8	1428.2	<0.0001	9.2	2.3	37.0	0.0045
	56	12	613.6	168.4	2235.6	<0.0001	15.4	3.6	65.3	0.0016
	84	5	2084.7	470.5	9236.9	0.0001	27.7	1.0	802.4	0.0519
	120	5	131.5	3.1	5594.9	0.0225	3.6	0.1	107.6	0.3594
	140	4	648.0	219.1	1916.0	0.0003	5.2	0.2	147.5	0.2147
	180	6	2156.3	1000.1	4649.3	<0.0001	16.2	0.8	349	0.0668
**NAb Omicron XBB.1.5**	0	13	12.9	7.4	22.2	<0.0001	NA	NA	NA	NA
	28	13	57.1	17.4	186.9	<0.0001	4.4	1.7	11.9	0.0062
	56	12	104.8	30.3	362.8	<0.0001	8.0	2.6	24.2	0.0017
	84	5	225.9	31.9	1599.4	0.0015	22.6	3.2	159.9	0.0115
	120	5	37.5	3.8	367.9	0.0116	2.0	0.2	16.7	0.4352
	140	4	301.2	83.7	1083.9	0.0008	30.1	8.4	108.4	0.0035
	180	6	256.5	80.5	817.3	1.00E-04	14.9	2.5	88.3	0.0114

***confidence intervals based on log10-transformed data following a Student's t distribution with N -1 degrees of freedom, NA = not applicable.**

Table S3. Cellular response geometric mean and geometric mean fold increase by study day

			Geometric Mean (GM)				Geometric Mean Fold Rise (GMFR)			
				95% CI				95% CI		
**Parameter**	Day	N	Est GM	LL	UL	p-value	Est GMFR	LL	UL	p-values
**IFNγ S**	0	13	124.1	43	357.7	<0.0001	NA	NA	NA	NA
	28	13	1576.7	386.3	6436.2	<0.0001	12.7	4.4	36.6	0.0002
	56	12	3691	1630.7	8354.3	<0.0001	25.9	9.4	71.4	<0.0001
	84	5	2651.1	978.5	7182.9	<0.0001	19.5	2.3	163.5	0.0177
	120	5	1717.4	385.2	7657	0.0002	15.0	2.0	113.4	0.0205
	140	4	3561.2	1726.8	7344.5	<0.0001	20.5	0.7	581	0.064
	180	6	4754.7	1735.2	13029	<0.0001	28.9	2.7	311	0.0149
**IFNγ N**	0	13	14.5	4.6	45.8	0.0003	NA	NA	NA	NA
	28	13	209.6	60.0	732	<0.0001	14.5	5.3	39.8	0.0001
	56	12	435.5	127.2	1491.3	<0.0001	28.3	9.6	83.1	<0.0001
	84	5	147.9	6.1	3612.9	0.0122	9.0	0.8	96.7	0.0618
	120	5	198.8	23.9	1654	0.0023	12.8	0.1	1132	0.1887
	140	4	380.6	46.6	3110.3	0.0029	15.2	0.9	261.9	0.056
	180	6	753.5	232.5	2441.2	<0.0001	31.5	4.4	225	0.0063
**IFNγ M**	0	13	9.9	3.0	32.8	0.0014	NA	NA	NA	NA
	28	13	9.2	2.4	34.3	0.0033	0.9	0.3	2.5	0.8756
	56	12	7.5	1.7	32.8	0.012	0.6	0.2	2.0	0.3867
	84	5	12.5	2.0	76.6	0.018	0.9	0.2	5.2	0.8644
	120	5	3.6	0.6	20.2	0.1124	0.5	0	18.3	0.6178
	140	4	21.6	0.9	538.3	0.0559	2.6	0.4	16.2	0.1974
	180	6	4.2	0.4	40.3	0.166	0.5	0.1	3.3	0.3872
**AIM S CD4**	0	9	0.3	0.1	1.1	0.0689	NA	NA	NA	NA
	28	8	3.8	1.5	9.5	0.0107	19.2	8.8	41.7	<0.0001
	56	8	6.4	3.5	11.5	0.0001	14.6	5.2	40.5	0.0005
	84	2	8.0	0.5	130.1	0.0671	7.2	0	Inf	0.5258
	120	5	5.5	2.9	10.3	0.0017	21.7	12.6	37.2	0.0001
	140	1	8.1	12.6	37.2	0.0001	1.1	NE	NE	NE
	180	3	5.2	0.3	86.8	0.1287	5.6	0.2	167.5	0.1619
**AIM N CD4**	0	9	0.0	0.0	0.3	0.0067	NA	NA	NA	NA
	28	8	1.7	0.4	7.1	0.4145	72.1	4.4	1190.6	0.0087
	56	8	3.0	1.0	8.5	0.0448	55.5	6.2	496.8	0.0034
	84	2	1.8	0.0	121.4	0.3163	69	0.0	Inf	0.3875
	120	5	2.0	0.6	6.3	0.1749	43.8	2.0	979.1	0.0278
	140	1	1.7	2.0	979.1	0.0278	2.5	NE	NE	NE
	180	3	1.2	0.0	50.6	0.8257	27.0	0.2	3200.6	0.0971
**AIM S CD8**	0	9	0.0	0.0	0.2	0.0036	NA	NA	NA	NA
	28	8	0.1	0.0	1.1	0.0591	4.3	0.8	23.2	0.0786
	56	8	0.7	0.2	1.9	0.3801	12.0	3.8	38.4	0.0015
	84	2	1.6	0.0	146538047.9	0.8087	11.2	0.0	160733487.4	0.3136
	120	5	0.3	0.1	1.4	0.1054	9.0	1.0	79.3	0.0479
	140	1	4.8	1.0	79.3	0.0479	2.2	NE	NE	NE
	180	3	0.7	0.0	65	0.7433	1.9	0.8	4.4	0.0904
**AIM N CD8**	0	9	0.0	0.0	0.0	0.0001	NA	NA	NA	NA
	28	8	0.0	0.0	0.2	0.0057	4.3	0.2	83.8	0.2786
	56	8	0.1	0.0	0.3	0.0012	13.0	4.3	38.9	0.0009
	84	2	0.0	0.0	18000730.1	0.2611	6.4	0.3	156.8	0.0855
	120	5	0.1	0.0	0.4	0.0113	6.8	0.2	270	0.2218
	140	1	0.1	0.2	270.0	0.2218	5.0	NE	NE	NE
	180	3	0.0	0.0	1.9	0.069	1.0	0.0	72.4	0.989

***confidence intervals based on log10-transformed data following a Student's t distribution with N -1 degrees of freedom, NA = not applicable, NE = not estimable**

Table S4. Comparison of COH04S1 HCT/CAR-T patient population with healthy adults vaccinated with COH04S1 and Comirnaty®.

	**COH04S1, HCT/CAR-T**	**COH04S1, HV**	**Comirnaty, HCW**	**COH04S1 HCT/CAR-T vs. COH04S1 HV** (p*)	**COH04S1 HCT/CAR-T vs. Comirnaty HCW** (p*)	**COH04S1 HV vs. Comirnaty HCW** (p*)
**Number of individuals, n**	13	9	16	/	/	/
**Age, mean (range)**	55 (38-73)	32 (25-41)	49 (24-74)	<0.0001	NS	0.0013
**Female, n (%)**	8 (62)	5 (56)	11 (69)	/	/	/
**Allogeneic HCT, n (%)**	6 (46)	/	/	/	/	/
**Autologous HCT, n (%)**	6 (46)	/	/	/	/	/
**CAR-T, n (%)**	1 (8)	/	/	/	/	/

*Calculated using two-tailed Mann-Whitney test, NS= p>0.05.

HCT=hematopoietic cell transplant, CAR-T=chimeric antigen receptor T cell, HV=healthy volunteers, HCW=healthcare workers, NS= not significant.

Table S5. Flow Cytometry Reagents

Reagent	Source (Brand)	Identifier (Catalog#)
Viability Dye L/D Near IR	Invitrogen	L34975
Anti-human CD3 FITC	Biolegend	300440
Anti-human CD4 Brilliant Violet 421	BD	566703
Anti-human CD8 Brilliant Violet 605	BD	301040
Anti-human CD137 APC	Biolegend	309810
Anti-human OX40 PE-Cy7	Biolegend	350012
Anti-human CD69 PE	BD	555531
Anti-human CD14 V500	Biolegend	561391
Anti-human CD19 V500	Biolegend	561121
Anti-human CD45RA PerCP	Biolegend	304156
Anti-human CCR7 PE/Dazzle 594	Biolegend	353236

Supplementary Figures

Figure S1

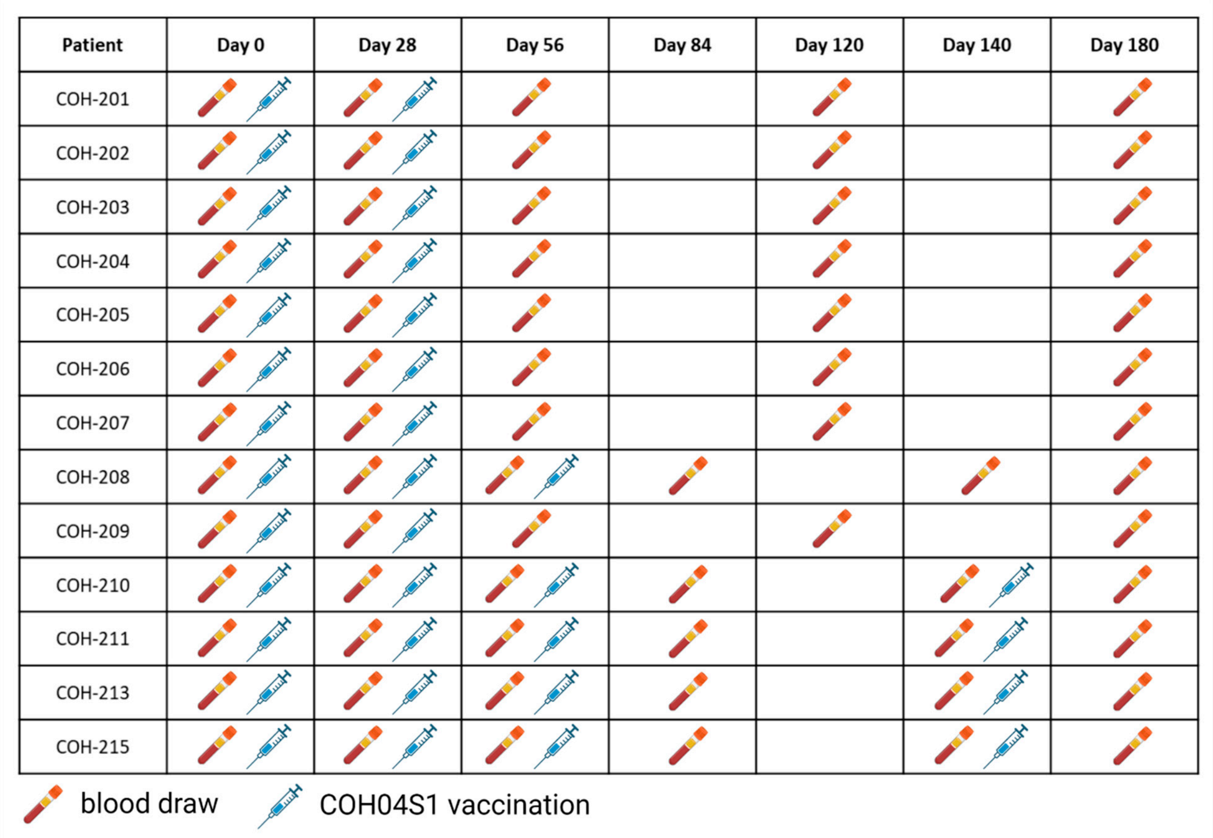

Patients' vaccination and sampling schedule. Patients (n=13) were vaccinated two-to-four times with COH04S1 at the indicated timepoints (syringes). Blood samples were collected at the indicated timepoint for immunological analyses (blood tubes). Created with BioRender.com.

Figure S2

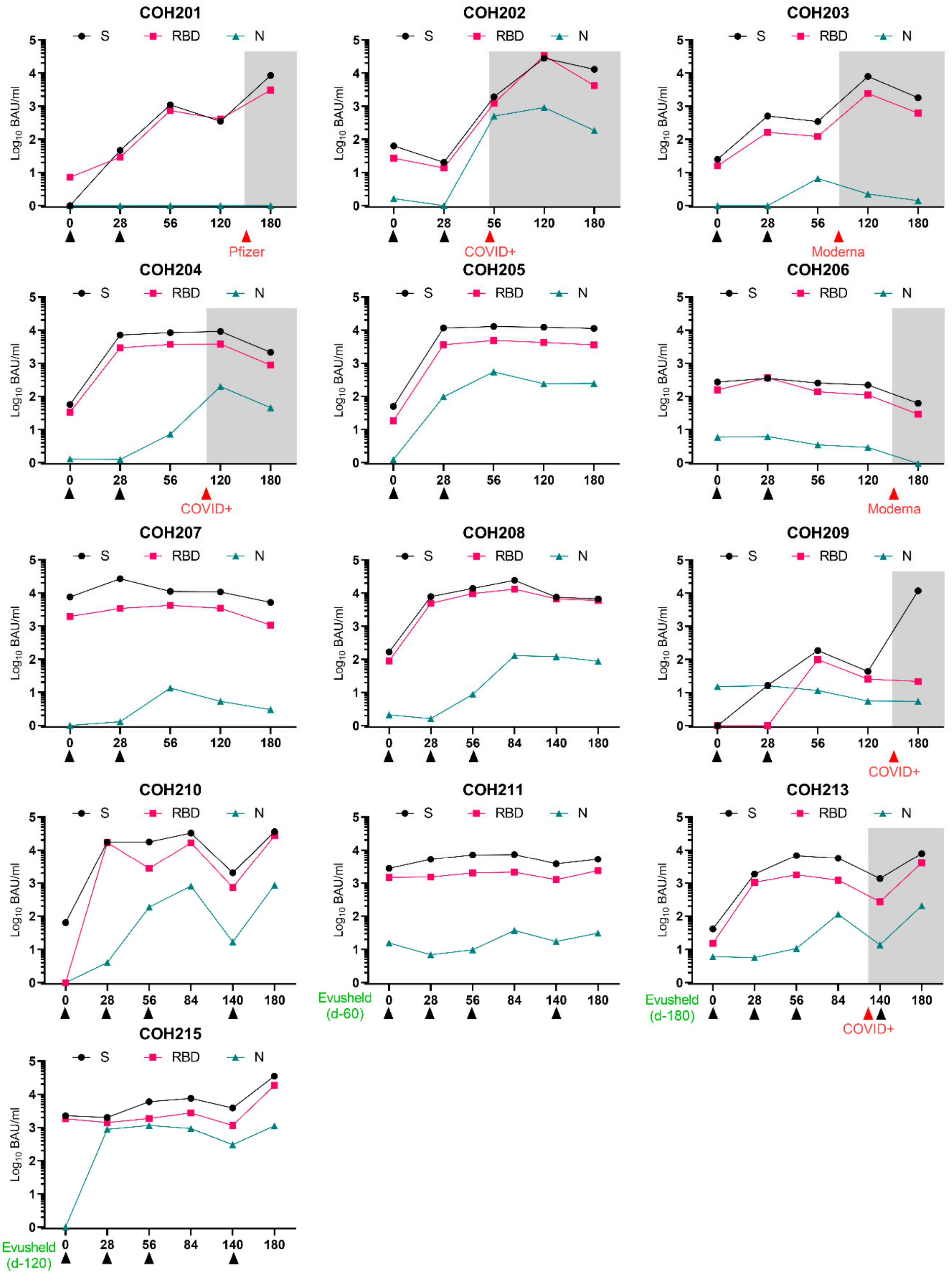

COH201
COH202
COH203
COH204
COH205
COH206
COH207
COH208
COH209
COH210
COH211
COH213
COH215
S
RBD
N
Log10 BAU/ml
Pfizer
COVID+
Moderna
Evusheld (d-60)
Evusheld (d-180)
Evusheld (d-120)

COH04S1 vaccine-elicited binding IgG in cancer patients post hematopoietic cell transplantation and cellular therapy. Patients (n=13) were vaccinated two-to-four times with COH04S1 (Figure S1), and serum samples were evaluated for SARS-CoV-2-specific binding IgG. Binding antibody titers to spike (S), receptor-binding domain (RBD), and nucleocapsid (N) antigens were measured by quantitative ELISA in serum samples at the indicated timepoints and expressed as binding antibody units (BAU)/ml. Black arrowheads indicate time of vaccination. Red arrowheads indicate COVID-19 positive test or vaccination with an FDA-approved mRNA vaccine from Moderna or Pfizer. Patients who received Evusheld® after transplant, but before COH04S1, are indicated in green, and the time of administration pre-COH04S1 vaccination is specified. Grey shadowing in the panels marks the samples that were excluded from the immunological analysis due to a disqualifying event (COVID-19+ test, or vaccination with COVID-19 approved vaccines Comirnaty® or Spikevax®).

Figure S3

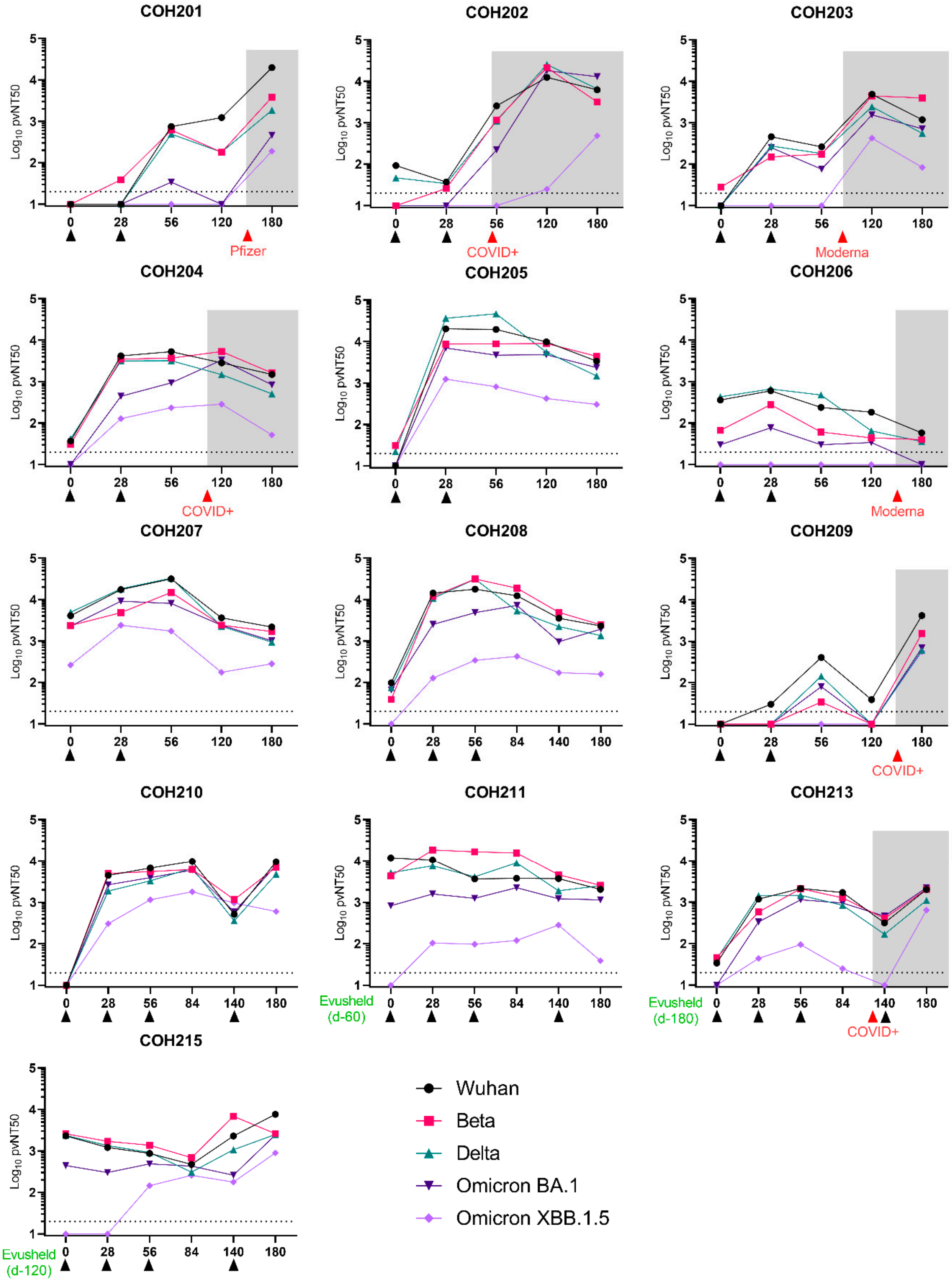

COH201
COH202
COH203
COH204
COH205
COH206
COH207
COH208
COH209
COH210
COH211
COH213
COH215
Log10 pvNT50
Pfizer
COVID+
Moderna
Evusheld (d-60)
Evusheld (d-180)
Evusheld (d-120)
Wuhan
Beta
Delta
Omicron BA.1
Omicron XBB.1.5

COH04S1 vaccine-elicited SARS-CoV-2 specific neutralizing antibodies (NAb) in cancer patients post hematopoietic cell transplantation and cellular therapy. Patients (n=13) were vaccinated two-to-four times with COH04S1 (Figure S1), and serum samples were evaluated for SARS-CoV-2-specific NAb responses against ancestral SARS-CoV-2 (Wuhan-Hu-1), Beta, Delta, and Omicron BA.1 variants at the indicated timepoints. 50% neutralizing antibody titers (NT50) were measured using a pseudovirus (PsV) assay. Dotted lines represent lower limit of detection. Black arrowheads indicate time of vaccination. Red arrowheads indicate COVID-19 positive test or vaccination with an FDA-approved mRNA vaccine from Moderna or Pfizer. Patients who received Evusheld® after transplant, but before COH04S1, are indicated in green, and the time of administration pre-COH04S1 vaccination is specified. Grey shadowing in the panels marks the samples that were excluded from the immunological analysis due to a disqualifying event (COVID-19+ test, or vaccination with COVID-19 approved vaccines Comirnaty® or Spikevax®).

Figure S4

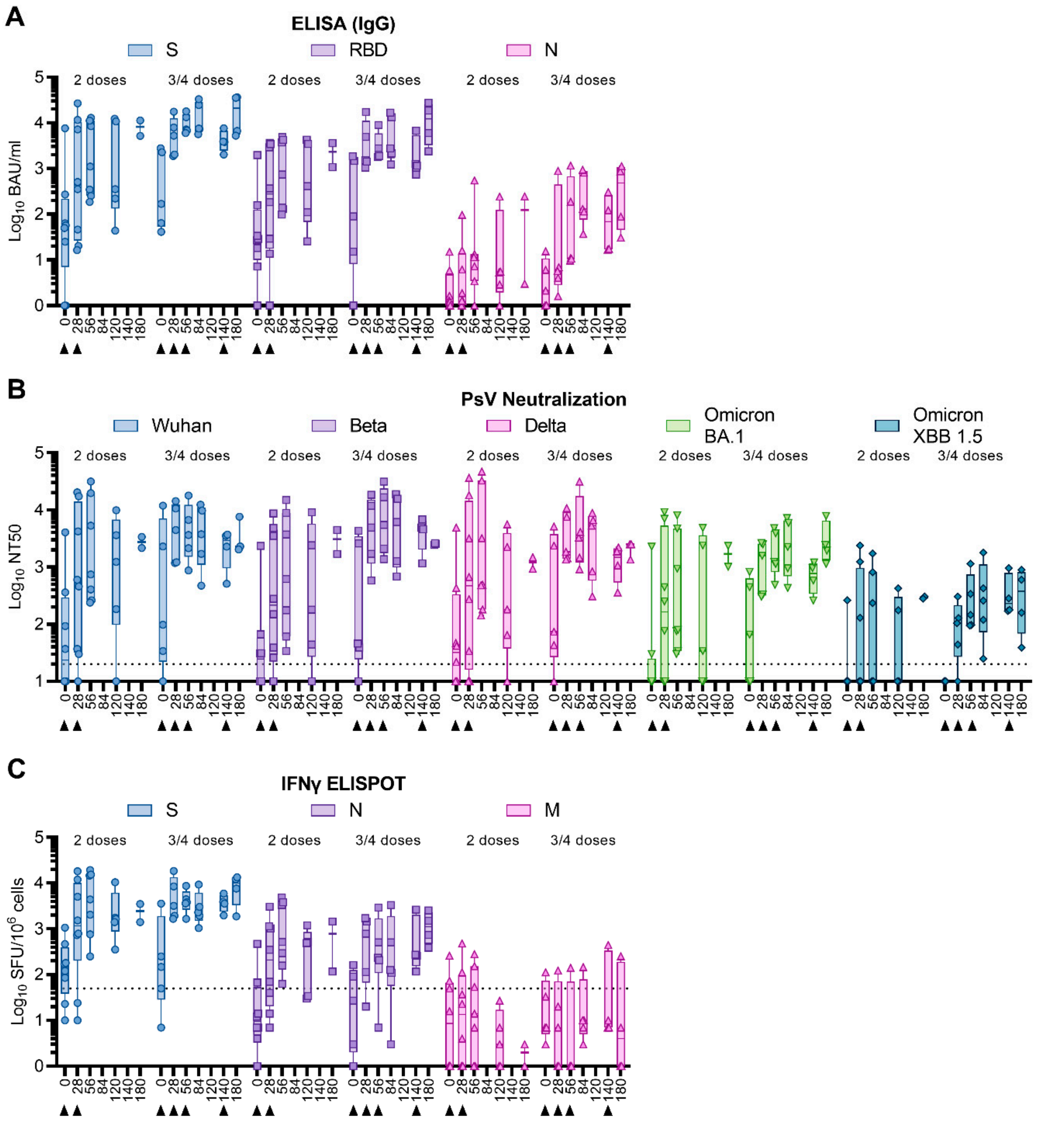

Comparison of humoral and cellular responses elicited by 2 or 3/4 COH04S1 immunizations. Cancer patients post hematopoietic cell transplantation and cellular therapy were vaccinated with two (n=8) or three/four doses (n= 5) of COH04S1 (Figure S1), and samples were evaluated for SARS-CoV-2-specific humoral and cellular immunity up to six months post-vaccination. A. Binding antibodies. Binding antibody titers to spike (S), receptor-binding domain (RBD), and nucleocapsid (N) antigens were measured by quantitative ELISA in serum samples at the indicated timepoints. B. NAb responses. 50% neutralizing antibody titers (NT50) against ancestral SARS-CoV-2 (Wuhan-Hu-1), Beta, Delta, and Omicron BA.1 and XBB .1.5 variants were measured using a pseudovirus (PsV) assay in serum samples at the indicated timepoints.

Dotted lines represent lower limit of detection. C. IFNγ T cells. T cell responses were quantified by IFNγ on PBMCs stimulated with S, N, and M peptide libraries at the indicated timepoints. Shown are the IFNγ spot forming units (SFU) measured in 10^6 PBMCs. Dotted lines represent the arbitrary threshold for a positive response (50 SFU/10^6 PBMCs). Data are presented as box plots extending from 25th to 75th percentiles, with lines indicating medians, and whiskers going from minimum to maximum values. Black arrowheads indicate time of vaccination.

Figure S5

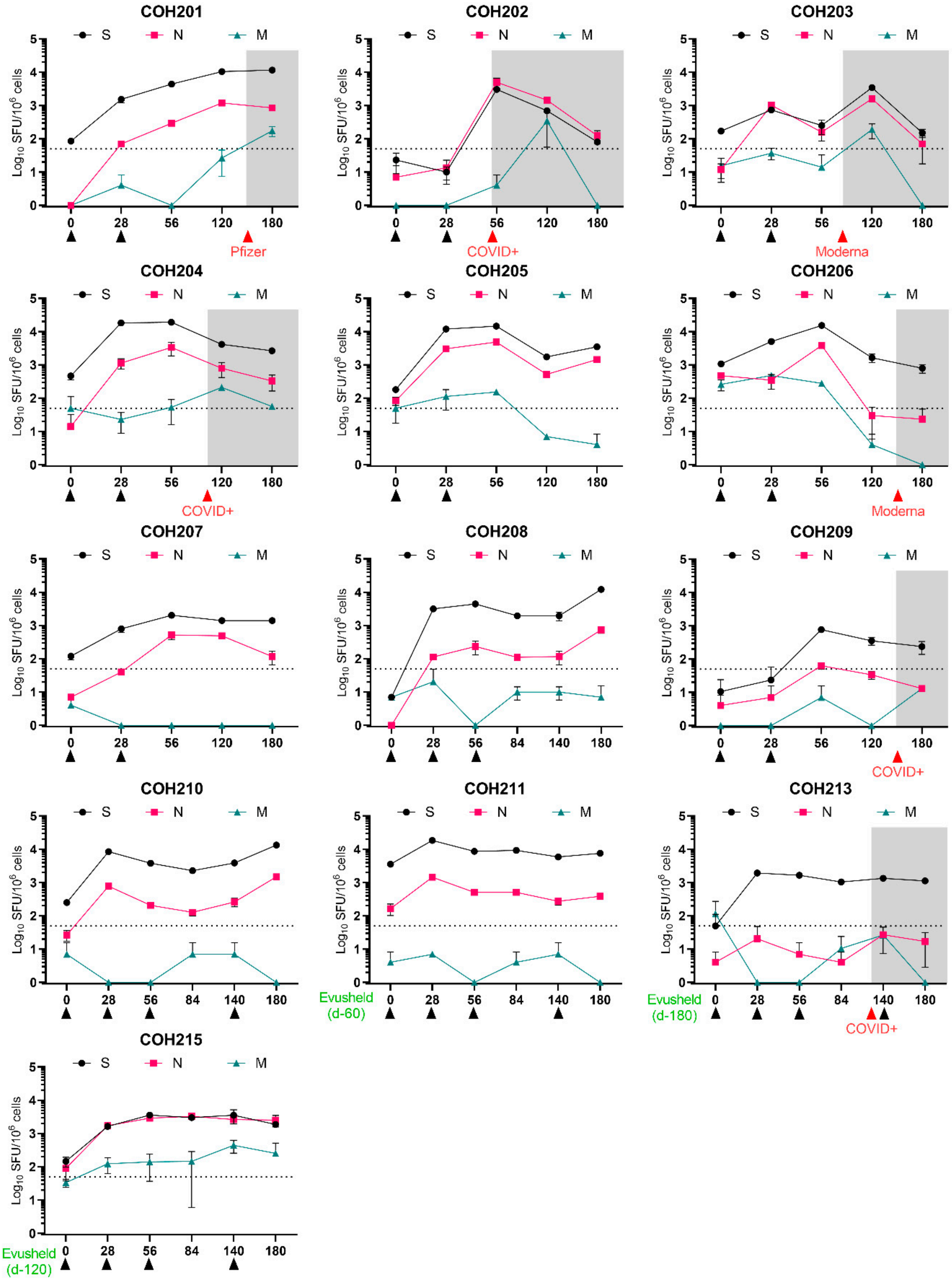
COH201
COH202
COH203
COH204
COH205
COH206
COH207
COH208
COH209
COH210
COH211
COH213
COH215
S
N
M
Log10 SFU/10^6 cells
Pfizer
COVID+
Moderna
Evusheld (d-60)
Evusheld (d-180)
Evusheld (d-120)

COH04S1 vaccine-elicited cellular immunity in cancer patients post hematopoietic cell transplantation and cellular therapy. Patients (n=13) were vaccinated two-to-four times with COH04S1 (Figure S1), and PBMCs samples were evaluated for SARS-CoV-2-specific cellular immunity. T cell responses were quantified by IFNγ on PBMCs stimulated with spike (S), nucleocapsid (N), and membrane (M) peptide libraries at the indicated timepoints. Shown are the IFNγ spot forming units (SFU) measured in 10^6 PBMCs. Black arrowheads indicate time of vaccination. Red arrowheads indicate COVID-19 positive test or vaccination with an FDA-approved mRNA vaccine from Moderna or Pfizer. Patients who received Evusheld® after transplant, but before COH04S1, are indicated in green, and the time of administration pre-COH04S1 vaccination is specified. Grey shadowing in the panels marks the samples that were excluded from the immunological analysis due to a disqualifying event (COVID-19+ test, or vaccination with COVID-19 approved vaccines Comirnaty® or Spikevax®).

Figure S6

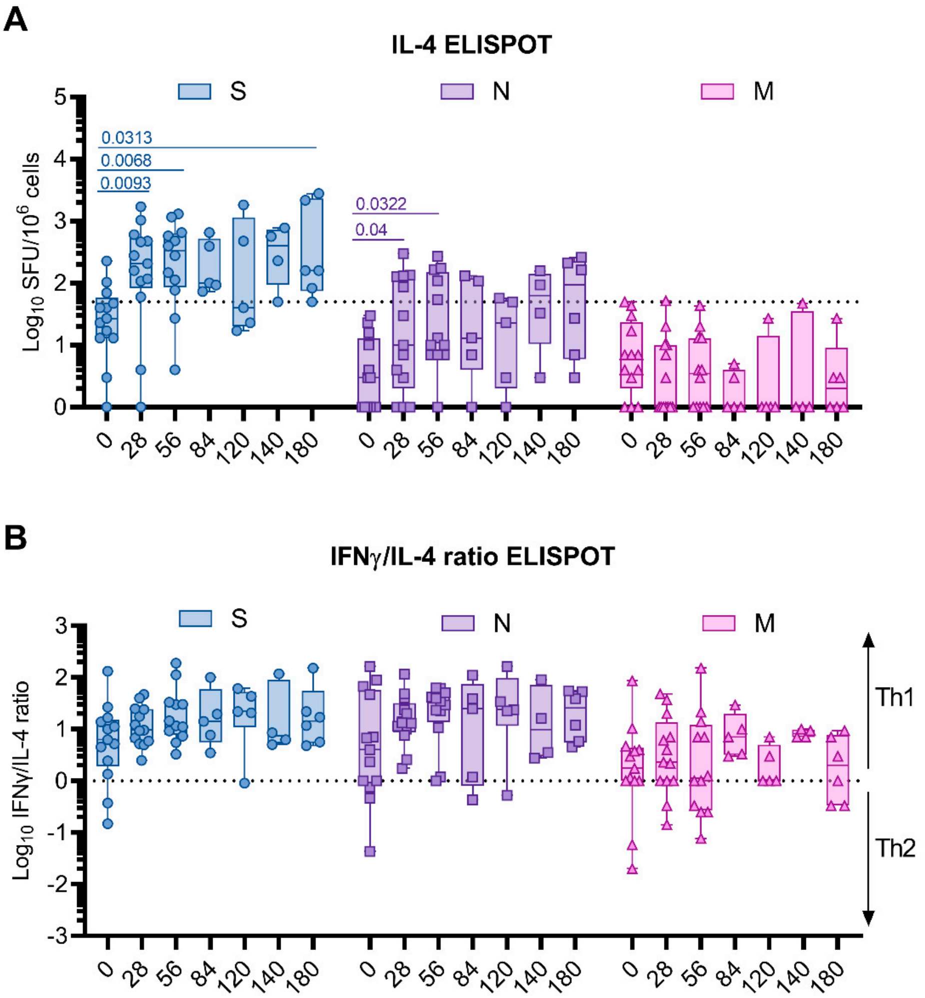

COH04S1-mediated cellular immunity in cancer patients post hematopoietic cell transplantation and cellular therapy. Patients (n=13) were vaccinated two-to-four times with COH04S1 (Figure S1), and PBMCs samples were evaluated for SARS-CoV-2-specific cellular immunity. A. IL-4 T cells. IL-4 secreting T cells were quantified by ELISPOT using PBMCs stimulated with S, N, and M peptide libraries at the indicated timepoints. Shown are the IL-4 spot forming units (SFU) measured in 10^6 PBMCs. Dotted lines represent the arbitrary threshold for a positive response (50 SFU/10^6 PBMCs). Wilcoxon signed-rank test (two-tailed) was applied to compare baseline to post-vaccine values. P values <0.05 are indicated. Where not indicated p>0.05. B. Th1/Th2 ratio. Ratios of S-, N-, and M-specific T cells secreting IFNγ and IL-4 were evaluated. Dotted line indicates a ratio of 1. A Th1-biased response is >1, a Th2-biased response is <1. Data are presented as box plots extending from 25th to 75th percentiles, with lines indicating medians, and whiskers going from minimum to maximum values.

Figure S7

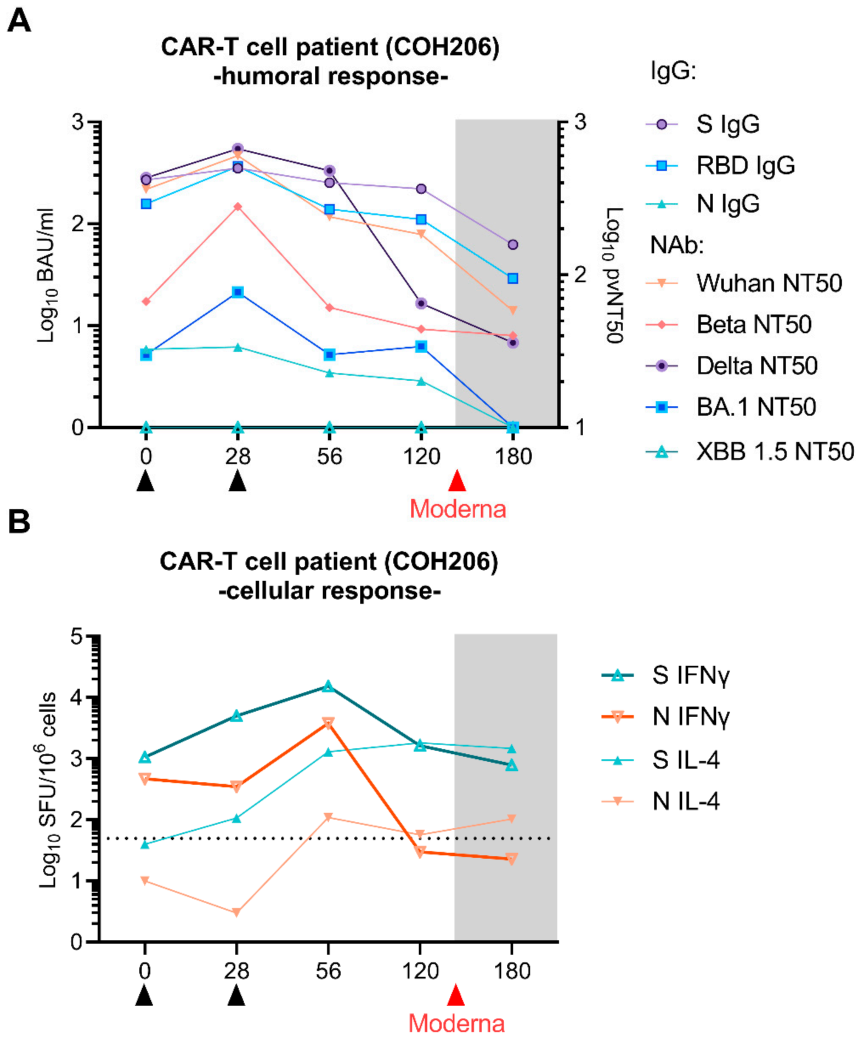

SARS-CoV-2-specific humoral and cellular immunity in CAR-T cell therapy patient COH206 vaccinated with two doses of COH04S1. CAR-T cell patient COH206 received two doses of COH04S1 (black arrowheads) about 8 months post treatment (Table S1). Blood samples were evaluated for SARS-CoV-2-specific humoral and cellular immunity up to six months post-vaccination. A. Humoral response. Binding antibody titers to spike (S), receptor-binding domain (RBD), and nucleocapsid (N) antigens were measured by quantitative ELISA in serum samples at the indicated timepoints. 50% neutralizing antibody titers (NT50) against ancestral SARS-CoV-2 (Wuhan-Hu-1), Beta, Delta, and Omicron BA.1 and XBB.1.5 variants were measured using a pseudovirus (PsV) assay in serum samples at the indicated timepoints. B. T cells. T cell responses were quantified by IFNγ/IL-4 ELISPOT on PBMCs stimulated with S and N peptide libraries at the indicated timepoints. Shown are the IFNγ/IL-4 spot forming units (SFU) measured in 10^6 PBMCs. Dotted line represents the arbitrary threshold for a positive response (50 SFU/10^6 PBMCs). Red arrowhead indicates time of vaccination with Spikevax® (mRNA-1273, Moderna). Grey shadowing marks the samples that were excluded from the immunological analysis.

Figure S8

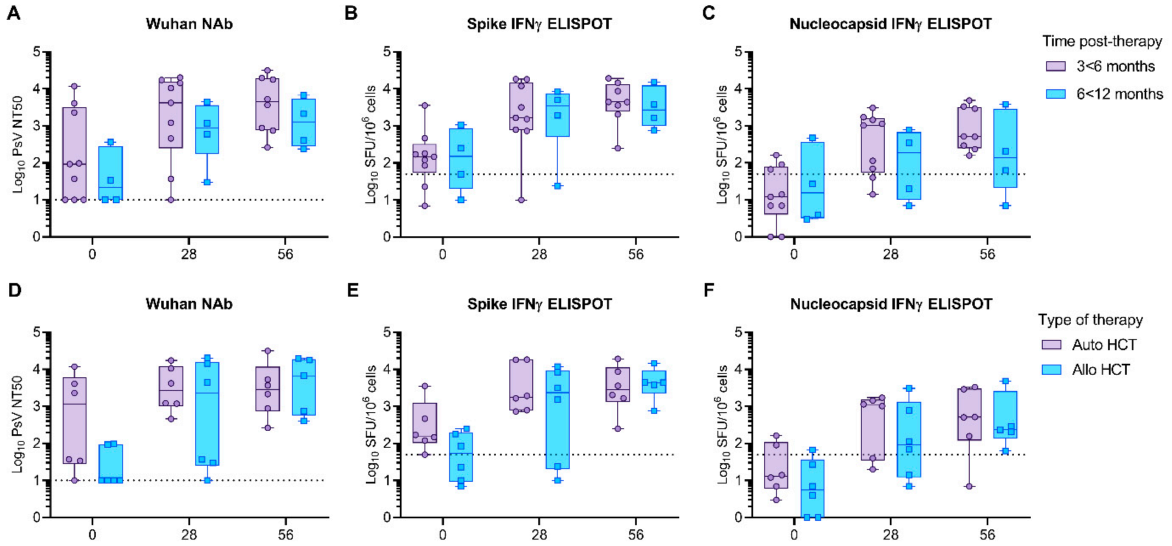

Differential immunogenicity of two doses of COH04S1 at early- and late-timepoints post-transplant and in auto-HCT and allo-HCT patients. Patients (n=13) were vaccinated two times with COH04S1, and blood samples were evaluated for SARS-CoV-2-specific humoral and cellular immunity. In A-C COH04S1 immunogenicity was compared between patients who received COH04S1 at 3-6- or 6-12-months post HCT/CAR-T. In D-F COH04S1 immunogenicity was compared between auto- and allo-HCT patients. A, D. NAb responses. 50% neutralizing antibody titers (NT50) against ancestral SARS-CoV-2 (Wuhan-Hu-1) were measured using a pseudovirus (PsV) assay in serum samples at the indicated timepoints. Dotted lines represent lower limit of detection. B-C, E-F. IFNγ T cells. S-specific (B, E) and N-specific (C, F) T cell responses were quantified by IFNγ on PBMCs stimulated with S and N peptide libraries at the indicated timepoints. Shown are the IFNγ spot forming units (SFU) measured in 10^6 PBMCs. Dotted lines represent the arbitrary threshold for a positive response (50 SFU/10^6 PBMCs). Data are presented as box plots extending from 25th to 75th percentiles, with lines indicating medians, and whiskers going from minimum to maximum values. Two-way ANOVA followed by Tukey's multiple comparison test was applied after log transformation. If not indicated p>0.05.

Figure S9

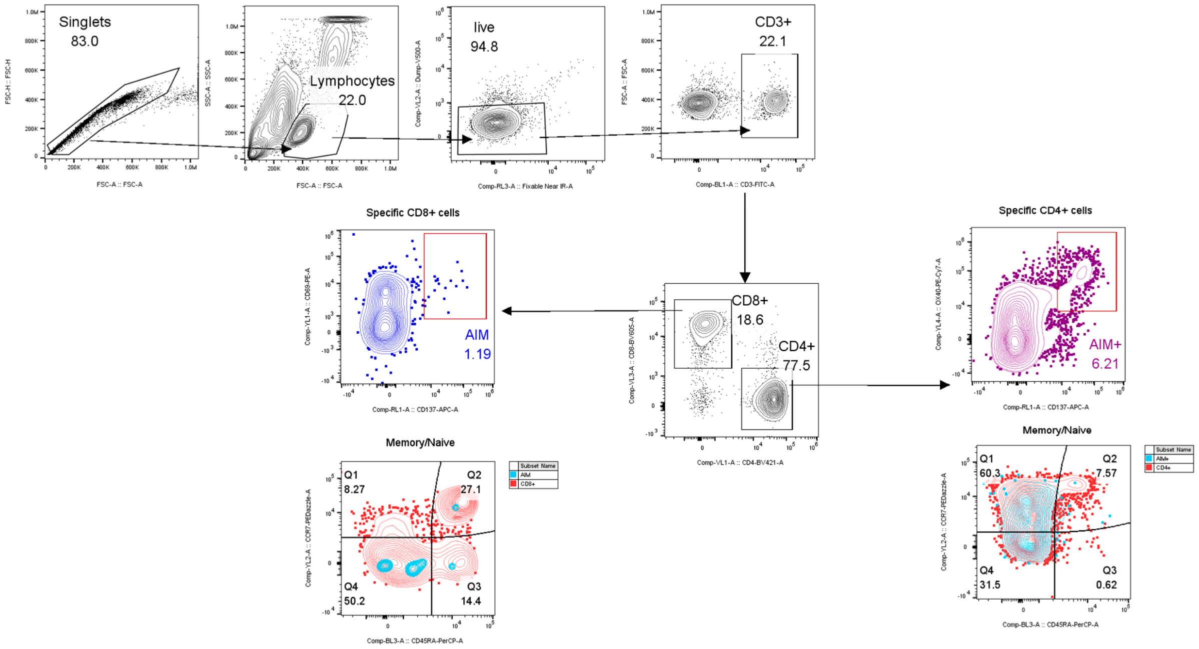

Gating strategy for the evaluation of S and N-specific T cells. The analysis was performed using the singlet cells, lymphocyte, and live cells (live and dead Near IR negative) gating. Then, cells were further gated for CD3+ followed by CD4+ and CD8+. From each population, the specific cells were evaluated by the expression of Activation Induced Markers (AIM+) CD137+, OX40+ for CD4+ T cells and CD137+, CD69+, for CD8+ T. Naive Vs Memory subsets were evaluated using CCR7 Vs CD45RA markers from total CD4+ and CD8+ (red) and from AIM+ (blue) cells. Q1=Central Memory (CCR7+, CD45RA-), Q2=Naive (CCR7+, CD45RA+), Q3=Terminally Differentiated Effectors (CCR7-, CD45RA+), Q4=Effector Memory (CCR7-, CD45RA-).

Figure S10

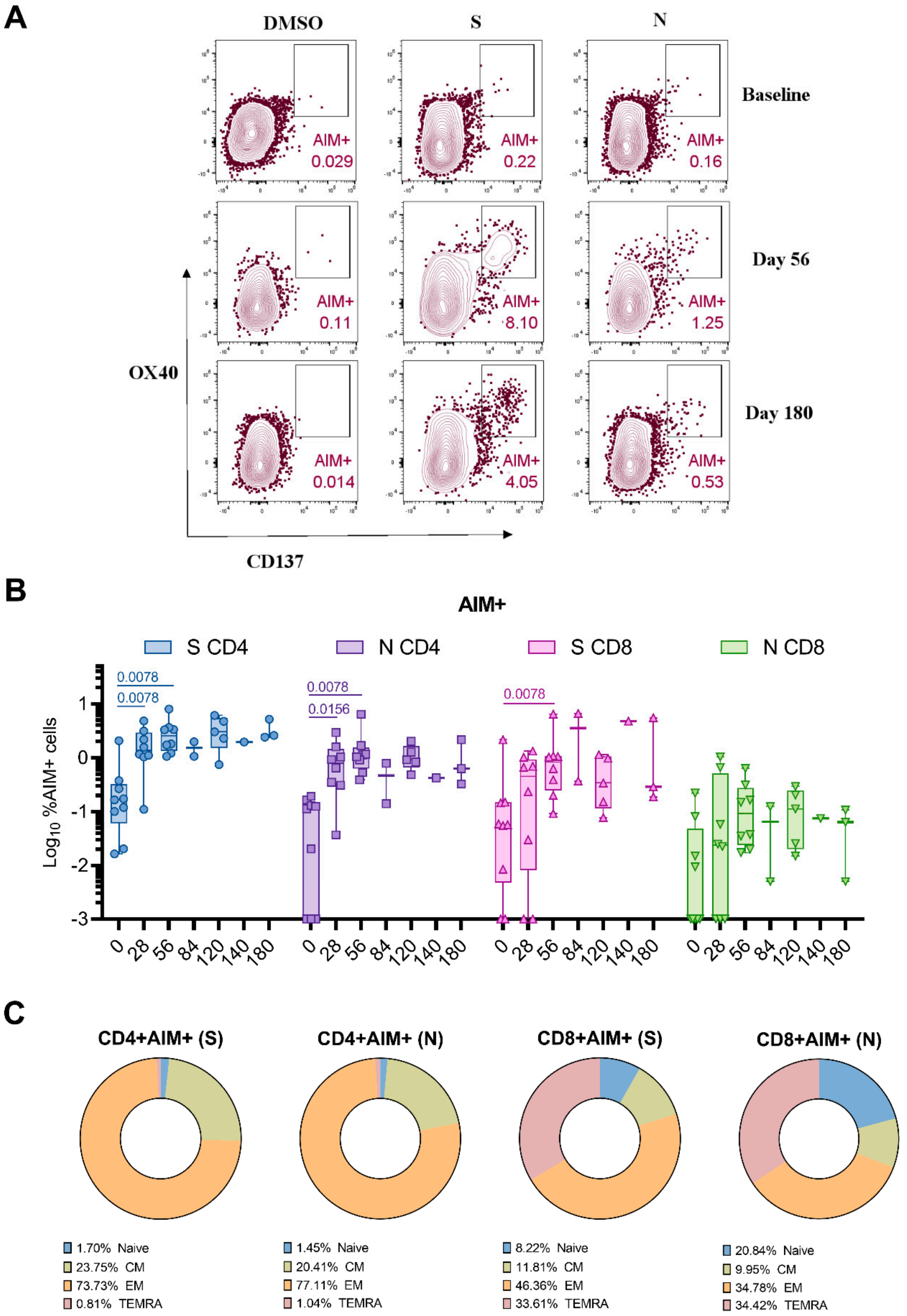
A
DMSO
S
N
Baseline
Day 56
Day 180
OX40
CD137
AIM+
0.029
0.22
0.16
0.11
8.10
1.25
0.014
4.05
0.53
B
AIM+
S CD4
N CD4
S CD8
N CD8
Log10 %AIM+ cells
0.0078
0.0078
0.0078
0.0156
0.0078
0 28 56 84 120 140 180
C
CD4+AIM+ (S)
CD4+AIM+ (N)
CD8+AIM+ (S)
CD8+AIM+ (N)
1.70% Naive
23.75% CM
73.73% EM
0.81% TEMRA
1.45% Naive
20.41% CM
77.11% EM
1.04% TEMRA
8.22% Naive
11.81% CM
46.36% EM
33.61% TEMRA
20.84% Naive
9.95% CM
34.78% EM
34.42% TEMRA

AIM assay and memory phenotype. A. Examples of S and N-specific CD4+ T cells. The specific CD4+ cells were evaluated by the expression of Activation Induced Markers (AIM+) CD137+, OX40+. Dot plot examples of negative control (DMSO), S-specific, and N-specific are shown for days 0, 56, and 180. B. Activation Induced Markers (AIM+) T cell percentage. Percentages of AIM+ T cells in PBMCs stimulated with S and N peptide libraries were quantified by cytofluorimetry at the indicated timepoints. Data are presented as box plots extending from 25th to 75th percentiles, with lines indicating medians, and whiskers going from minimum to maximum values. Wilcoxon signed-rank test (two-tailed) was applied to compare baseline to post-vaccine values. P values <0.05 are indicated above the figures. Where not indicated $p>0.05$. C. T cells memory phenotype. Pie charts show T cells memory phenotypes at one-month post second COH04S1 dose. Naive/Memory subtypes were evaluated in S and N-specific CD4+ and CD8+ T cells. Mean percentage of population is shown. In all pie charts n= 9 with the exclusion of CD8+AIM+(N) where n=3. CM=central memory, EM=effector memory, TEMRA=EM cells re-expressing CD45RA.

Figure S11

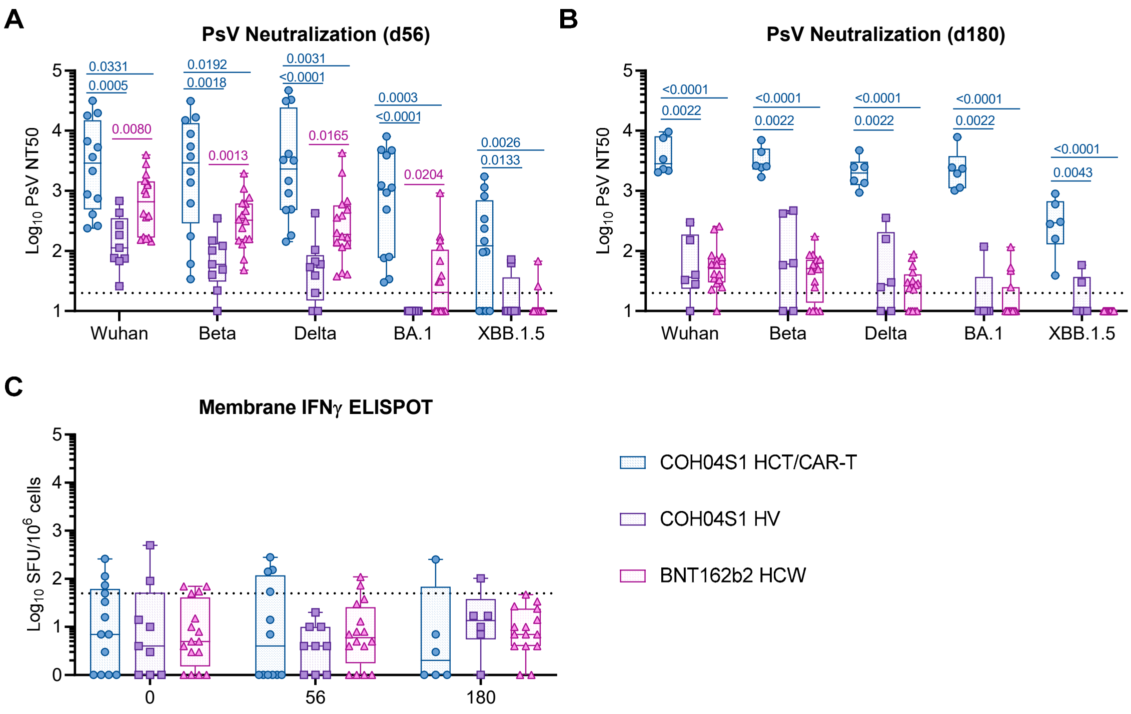

Immunogenicity of COH04S1 in HCT/CAR-T patients and COH04S1 or Comirnaty® in healthy adults. (A-B) SARS-CoV-2 neutralizing antibody titers to SARS-CoV-2 Wuhan-Hu-1 ancestral strain and Beta, Delta, and Omicron BA.1 and XBB.1.5 variants were measured in samples of COH04S1-vaccinated HCT/CAR-T patients at days 56 (A, n=12) and 180 (B, n=6) post-vaccination and compared to responses measured in healthy volunteers (HV, n=9 d56, n=6 d180) vaccinated with COH04S1 or healthcare workers (HCW, n=17) vaccinated with FDA-approved mRNA vaccine Comirnaty® at the same timepoint. Dotted lines in A-B represent lower limit of detection. In B 4/6 HCT/CAR-T patients had received three/four COH04S1 doses. (C) IFNγ T cells specific for SARS-CoV-2 membrane (M) were measured at baseline, day 56 and 180 post-vaccination in the same subjects as in A-B. Dotted line represents the arbitrary threshold of positivity (50 spots/10^6 cells). Data are presented as box plots extending from 25th to 75th percentiles, with lines indicating medians, and whiskers going from minimum to maximum values. Values were compared using two-tailed Mann-Whitney test. P values <0.05 are indicated. Where not indicated p>0.05.
